# Crystal Structure of 70S Ribosome with Both Cognate tRNAs in the E and P Sites Representing an Authentic Elongation Complex

**DOI:** 10.1371/journal.pone.0058829

**Published:** 2013-03-19

**Authors:** Shu Feng, Yun Chen, Yong-Gui Gao

**Affiliations:** 1 School of Biological Science, Nanyang Technological University, Singapore; 2 Institute of Molecular and Cell Biology, Proteos, Singapore; 3 MRC Laboratory of Molecular Biology, Cambridge, United Kingdom; University of Lethbridge, Canada

## Abstract

During the translation cycle, a cognate deacylated tRNA can only move together with the codon into the E site. We here present the first structure of a cognate tRNA bound to the ribosomal E site resulting from translocation by EF-G, in which an entire L1 stalk (L1 protein and L1 rRNA) interacts with E-site tRNA (E-tRNA), representing an authentic ribosome elongation complex. Our results revealed that the Watson-Crick base pairing is formed at the first and second codon-anticodon positions in the E site in the ribosome elongation complex, whereas the codon-anticodon interaction in the third position is indirect. Analysis of the observed conformations of mRNA and E-tRNA suggests that the ribosome intrinsically has the potential to form codon-anticodon interaction in the E site, independently of the mRNA configuration. We also present a detailed description of the biologically relevant position of the entire L1 stalk and its interacting cognate E-tRNA, which provides a better understanding of the structural basis for translation elongation. Furthermore, to gain insight into translocation, we report the positioning of protein L6 contacting EF-G, as well as the conformational change of the C-terminal tail of protein S13 in the decoding center.

## Introduction

Proteins are synthesized by the ribosome in a process called translation. In bacteria, translation comprising initiation, elongation, and termination, involves four GTPase factors, initiation factor 2 (IF2), elongation factors Tu and G (EF-Tu/G), and peptide release factor 3 (RF3), respectively, reviewed in [1]. In particular, EF-Tu and EF-G perform the coordination role in the elongation cycle, which is at the heart of translation. EF-Tu delivers aminoacyl tRNA to the ribosomal A site, then peptidyl transfer from the P to the A site occurs, resulting in a pretranslocational state where the ribosome has a deacylated tRNA in the P site and a peptidyl-tRNA in the A site. Subsequently, ribosome would spontaneously adopt a ratcheted conformation (rotation by ∼6° of the 30S and 50S subunits relative to each other) [2], in which the aminoacyl ends of both tRNAs move into the P and E sites in the 50S subunit whereas anticodon stem-loops (ASLs) still remain in the A and P sites in the 30S subunit [3]. The binding of EF-G•GTP to ribosome in this ratcheted state (also called hybrid state) catalyzes translocation, which involves movement of the ASLs of tRNAs and the mRNA with respect to 30S subunit leading to the posttranslocational state where the ribosome preserves a peptidyl-tRNA and a deacylated tRNA in the P and E sites, respectively. The hydrolysis of EF-G•GTP to EFG•GDP is able to accelerate this step [4]. After GTP hydrolysis, the conformational change of EF-G renders it incompatible with ribosome binding, resulting in a rapid release. However, in the presence of the antibiotic fusidic acid, EF-G can be trapped in the ribosome [5].

During the aforementioned translation elongation cycle involving EF-Tu and EF-G, the tRNAs pass through the three ribosomal sites (A, P, and E). Of the three tRNA-binding sites, the E site was initially proposed by Nierhaus and his colleagues [6]. The location of the E site on ribosome was first visualized by low-resolution cryo-electron microscopy (Cryo-EM) [7], later studied by X-ray structure analysis [8]. The E site had been implicated in several activities, such as hybrid state formation and translocation [9], [10], translational fidelity of aminoacyl-tRNA selection leading to a more stringent decoding by negatively cooperation of “E/A sites” [11], and maintenance of mRNA reading frame [12]. However, for certain function of the ribosomal E site, particularly the coupling role between tRNA-binding events in the E and A sites [13], [14], is still a matter of controversy. Therefore, structural information on a cognate E-tRNA bound to the ribosome could be considerably helpful to clarify the function of E site.

Since a cognate tRNA accompanies the mRNA codon moving into the E site during translocation, so it is generally agreed that codon-anticodon pairing would occur in the E site. However, the kinetic stability of E-tRNA, as well the degree to which codon-anticodon interaction contributes to E-tRNA binding remain to be established. The structure of ribosome with mRNA and modified tRNA (*E. coli* tRNA^Phe^_GAA_ containing hypermodified nucleotide ms^2^i^6^A37) resembling post-initiation complex was recently reported [15], and reveals codon-anticodon pairing only for the first position in the E site, consistent with their previous structure at low resolution 5.5 Å [16]. As pointed by the authors [15], whether the base pair could exist without stabilization by the tRNA modification is uncertain. Therefore, to what extent base pair is formed for the three bases in E site remains elusive. A major obstacle is because of non-specific binding of tRNAs to E site. For structural studies, the incubation of 70S ribosome complex with large excess of tRNA leads to non-cognate tRNA binding to the E site. As a result, a non-cognate tRNA or a mix of tRNAs not engaging in codon-anticodon interaction in the E site has often been observed [11], and the significance of the observed stacking between the cognate tRNA and the L1 stalk also remains unclear.

To address the question of base pairing between mRNA and tRNA in the E site, we prepared the 70S ribosome with tRNA^Phe^ and tRNA^fMet^ bound in the A and P sites, this complex was subsequently incubated with EF-G which catalyzes translocation. The resulting ribosome complex with cognate tRNAs bound to both P and E sites (that came originally from the A and P sites), represents an authentic elongation complex in the posttranslocational state. We used this complex for crystallization trial, and finally determined the crystal structure at 3.7 Å resolution. The unbiased difference Fourier electron density (F_O_–F_C_) map clearly showes that tRNA is specifically bound to the E site forming base pairing with the mRNA codon, structurally proving the presence of an authentic ribosome elongation complex. In addition, we obtained the structure of a complete L1 stalk, comprising L1 protein and L1 rRNA, as well as the interacting cognate E-tRNA. Thus, the biologically relevant position of these three components and the interacting E-site codon (E-codon) was revealed in detail, which is of critical importance for a better understanding of the structural basis for translation elongation. Moreover, the description of the positioning of protein L6 contacting with EF-G in ribosome, and the observation of conformational change of the C-terminal tail of protein S13 in the decoding center could allow us to rationalize the relevant biochemical data and provide insights into translocation. These features with regard to L6 and S13 could be similarly observed in the previous structure [17], but have not yet been reported.

## Materials and Methods

### Protein, Ribosome, tRNA, and mRNA

*Thermus thermophilus* EF-G was cloned, expressed, and purified with the same procedure as previously described [17]. 70S ribosomes harboring a C-terminal truncation of protein L9, *Escherichia coli* tRNA^fMet^, and tRNA^phe^ (both deacylated) were prepared using the previously described method [18], [19]. The mRNA Z4C was chemically synthesized (Dharmacon) with the sequence: 5′ GGCAAGGAGGUAAAA**AUGUUC**AAAA 3′, with an fMet codon at the P site (bold) and a Phe codon at the A site (underlined bold).

### Complex Formation

70S ribosome at a final concentration of 4.0 µmol/L and 8.0 µmol/L mRNA was incubated in buffer G with low concentration of magnesium (5 mM HEPES pH 7.5, 50 mM KCl, 10 mM NH4Cl, 4.5 mM Mg-acetate) at 55°C for 6 min, then 16.0 µmol/L tRNA^fMet^ was quickly added and the complex was incubated at 55°C for 30 min, subsequently 16.0 µmol/L tRNA^Phe^ was added and incubated at 55°C for another 30 min. Simultaneously, a final concentration of 500 µmol/L fusidic acid, 20 µmol/L EF-G and 100 µmol/L GTP which had been mixed and pre-incubated at room temperature for 30 min, were added into the ribosome complex, and the resulting complex was incubated for 30 min at 55°C and at room temperature for 30 min prior to crystallization. Deoxy Big Chap (DOBC, Hampton Research) was added to the complex and magnesium concentration was simultaneously raised to 10 mM, resulting in the final concentration of 3.3 µmol/L 70S ribosome and 2.3 µmol/L DOBC, respectively.

### Crystallization, X-ray Data Collection, and Structure Determination

Based on the crystallization condition as previously reported [17], several rounds of optimization were performed. Finally, crystals were grown in sitting-drop trays by mixing 3 ul reservoir (0.1 M MES pH 6.6, 8.5–9.0% PEG 20 K, 0–25 mM KCl) with 3 ul ribosome complex. Crystals grew to full size within two weeks, after stepwise cryoprotection to a final concentration of 25% PEG 400 in the mother solution, crystals were then frozen by plunging into liquid nitrogen.

Diffraction data were collected at 100 K on beamline of PXI of the Swiss Light Source (SLS), and all data were processed with XDS [20]. Using the empty 70S ribosome [17] as an initial model, refinement with CNS [21] was carried out and difference density map clearly revealed the presence of the mRNA and tRNA ligands. All model building was done using COOT [22], and electron density map was generated with CNS [21] or CCP4 suite [23]. The coordinates and structure factors have been deposited in Protein Data Bank (PDB) with accession IDs 4B8F, 4B8G, 4B8H, and 4B8I. Crystallographic data and refinement are summarized in Table 1. All figures were made with PyMOL (DeLano Scientific).

**Table 1:** Summary of crystallographic data and refinement statistics.

Data collection	
Space group	P2_1_
Unit cell dimentions	
a,b,c (Å)	a = 291.4, b = 269.4, c = 401.9
α,β,γ (°)	α = γ = 90, β = 91.8
Resolution (Å)	50–3.7 (3.8–3.7)
R_sym_ (%)	20.5 (108.0)
I/σI	8.2 (1.64)
Completeness (%)	99.8 (99.9)
Redundancy	7.3 (6.1)
**Refinement**	
Resolution (Å)	50–3.7
No. of unique reflections	657561
Rwork/Rfree	22.2/26.7
No. of atoms	
RNA	200844
Protein	100104
Average B factor	
RNA	92
Protein	103
Rmsd from idealty	
Bond length (Å)	0.007
Bond angle (°)	1.2

* Numbers in parenthesis refer to outer resolution shell.

† I/σ = 2.0 at 3.75 Å.

## Results

### 1. Codon-anticodon Interaction in the Ribosomal E Site

**Figure 1:**
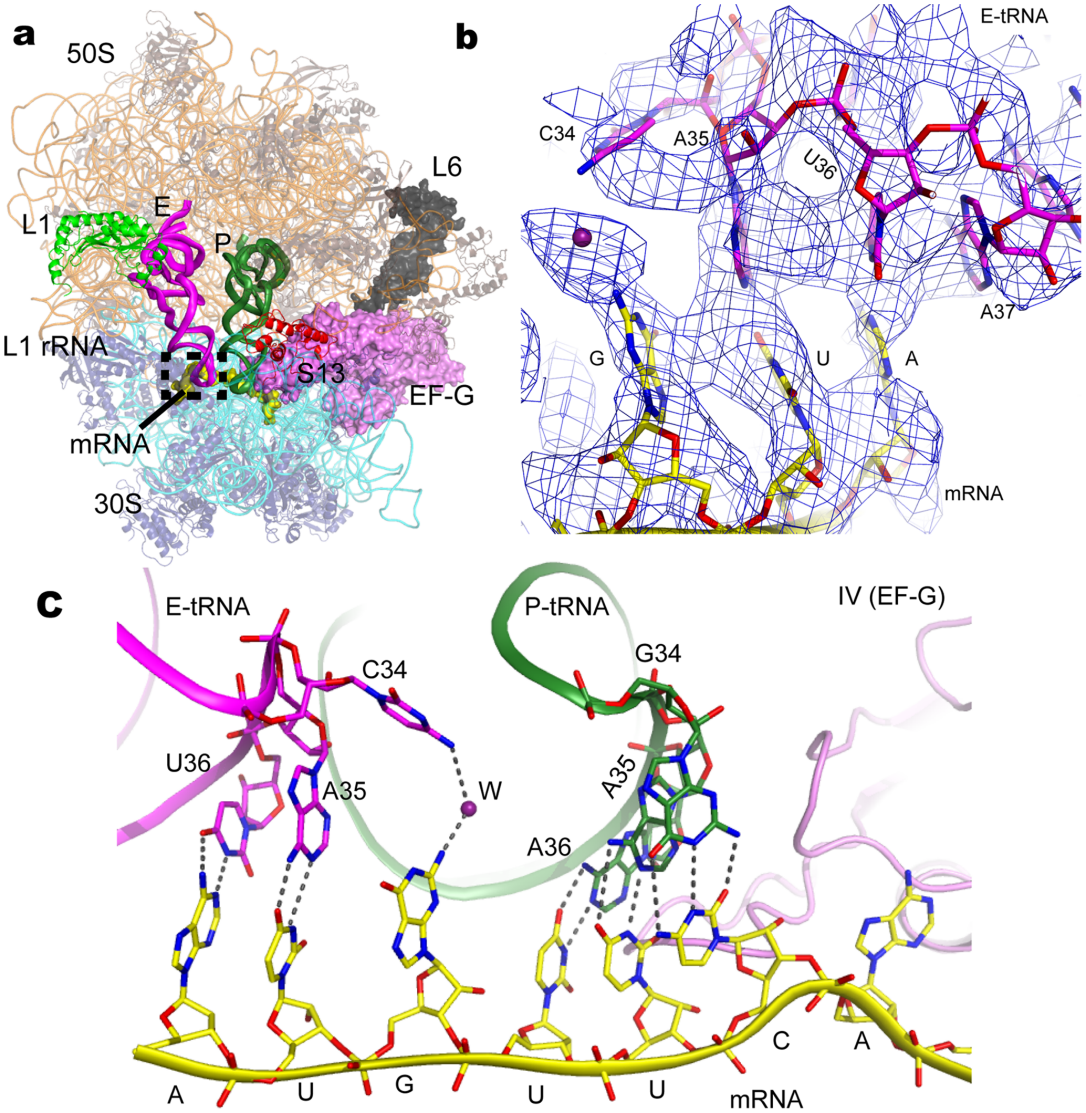
Overall structure of 70S ribosome with a cognate tRNA and codon-anticodon interaction in the E site. (a). Overall structure of two cognate tRNAs (P and E sites) bound to 70S ribosome complexed with EF-G representing an authentic posttranslocational state. EF-G, colored violet (same as below), is represented as surface model. Three ribosomal proteins, L1, L6, and S13, that will be described in the text, colored green, black (surface show), and red, respectively, are labeled in the overall structure. The codon-anticodon in E site is indicated by dashed rectangle. (b). Unbiased difference Fourier electron density map displayed at 1.2σ with refined E-site mRNA and tRNA. Based on the map, one water molecule was located and shown as firebrick sphere. (c). Interactions of mRNA and tRNA in both P and E sites. The dashed lines indicate hydrogen bonds, and W represents one water molecule.

We here report the crystal structure of the 70 S ribosome with an entire L1 stalk and its interacting cognate E-tRNA, which is originally translocated from the P site with the aid of EF-G (Fig. 1A). The unbiased difference F_O_–F_C_ map demonstrates that tRNA is specifically bound to E site with respect to the mRNA codon (Fig. 1B), and consequently the structure represents an authentic ribosome elongation complex, comprising two cognate tRNAs and EF-G trapped by fusidic acid in the posttranslocational state. This completes the previous structure (PDB: 2WRI and 2WRJ) which presents EF-G bound to the ribosome with a cognate P-tRNA and a non-cognate E-tRNA [17]. Although the overall structures of the two complexes are quite similar, contrary to the previous structure, we remarkably observed the interactions between codon and anticodon in the E site, and therefore provides new information on the function of the E site.

As depicted in Figure 1C, the first nucleotide A of mRNA codon in the E site, forms a base pair with the nucleotide U36 of tRNA^fMet^ anticodon, in a similar manner as observed by Jenner et al [16]. The central nucleotide U in the triple E-codons makes a clearly visible base pairing with the anticodon A35 of tRNA which has good fit with the unbiased difference F_O_–F_C_ map (Fig. 1B and 1C). This base pair is observed for the first time in ribosome structure. The base of the third codon G, stacks nicely with the central base U, however it is too far away to make a direct interaction with its anticodon C34 since C34 base flips out (Fig. 1C). Interestingly, it appears that one water molecule is located between the third codon G and the anticodon C34, forming a network of interaction involving bilateral hydrogen bonds with the bases of codon G and anticodon C34, respectively. The codon-anticodon interaction, particularly the non-base pair at the third position, is relevant to that observed for the A site in which the third (“wobble”) position of the codon is free to accommodate a certain noncanonical base pair [24]. This feature to a certain extent provides a structural hint for a coupling connection between the A and E sites, taken together to rationalize the degeneracy of the genetic code [11]. Indeed, recent observations suggested that the quality of codon-anticodon interactions in the E site might impact the interactions with both aminoacyl-tRNA and release factor substrates in the A site [25]. It is likely that a cognate tRNA occupies the ribosomal E site structurally supported by these specific codon-anticodon interactions, although the resolution of our electron density map does not permit to distinguish between the tRNA^fMet^ and tRNA^Phe^ because of the similarity of their primary sequences.

There are several types of decoding errors in the process of protein translation [11]. The most fatal is the frameshift as it causes loss of the correct reading frame, resulting in a dysfunctional, even toxic protein. Ribosome has evolved many features to prevent frameshift occurring [26]. A cognate tRNA in the E site is also pivotal for maintaining the mRNA reading frame, and likely can be ascribed to codon-anticodon interaction reported here (Fig. 1).

### 2. Conformations of mRNA and tRNA Upon Establishing Codon-anticodon Interaction in the E Site

**Figure 2:**
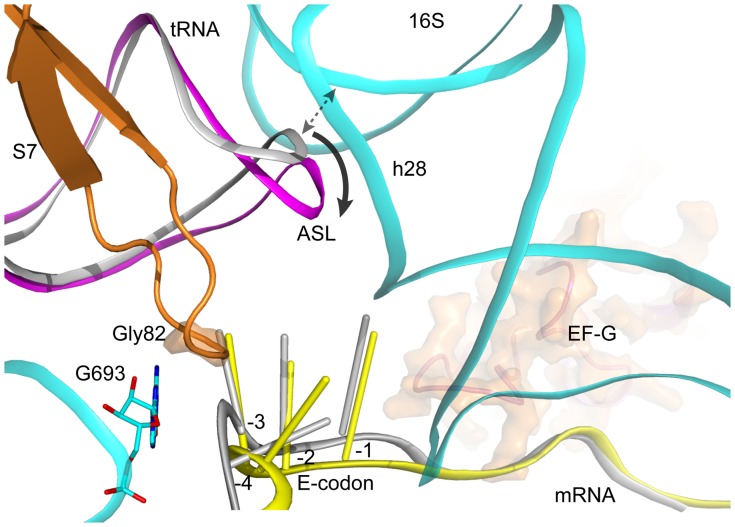
Close-up view of ribosomal elements around E codon and the conformational change. RNA helices are numbered with the standard Brimacombe nomenclature, prefixed by H for 23S rRNA and h for 16S rRNA, and RNA residues are numbered with the *E. coli* sequence throughout this paper. Except for non-cognate tRNA and mRNA colored grey that are taken from our previous structure (PDB 2WRI), other components, 16S rRNA (colored cyan), S7 (colored orange), tRNA and mRNA are presented in the present ribosome complex. The E codon and the immediately upstream nucleotide are shown as stick, are labeled by −4 to −1 based on the position related to the first nucleotide A (position +1) in the original P codon AUG of the mRNA Z4C. Upon establishing codon-anticodon interactions, ASL shifts by ∼5 Å, apart from h28 of 16S rRNA, resulting in disruption of ASL interaction with 16S rRNA, indicated by double-headed arrow. Both tRNA anticodons in the two structures points towards mRNA codon, therefore are not shown.

Upon establishing codon-anticodon interaction in the E site, the tip of ASL shifts towards the codon by approximately 5 Å compared with the previous ribosome structure with a non-cognate E-tRNA [17], although the other parts of both tRNAs occupy similar positions (Fig. 2 and Fig. S1). The interaction of cognate E-tRNA with mRNA is enhanced, which is extremely important for its role in maintenance of the translational reading frame. By contrast, it appears that the tip of ASL of cognate E-tRNA moves away from h28 of 16S rRNA, disrupting the interaction of non-cognate tRNA with h28 as was observed in the previous ribosome complex [17] (Fig. 2). Notably, an intermediate state of ASL was unravelled for the structure of post-initiation complex [16] (Fig. S1). Taking into account that Watson-Crick base pair was established only at the first position of E-codon, it is likely that the tendency of ASL to shift towards the E-codon, and move away from h28 of 16S rRNA, is consistent with the extent of forming codon-anticodon interaction. Thus, we propose that the energetic association of cognate E-tRNA with ribosome during translation is critical to keep the two sides balanced: stable binding with the mRNA codon to enhance frame maintenance; whereas weak interaction with 16S to accelerate the process for tRNA itself release.

It was postulated that the mRNA bound to the ribosome could be in two forms, “tensed” and “relaxed” depending on the distance between the core adenosine (−8) of the Shine-Dalgarno (SD) sequence and the first P codon (+1), and mRNA in “relaxed” form is favorable for the formation of codon-anticodon interaction at the ribosomal E site [15]. We previously reported the structure of ribosome which preserves a vacant tRNA in the ribosomal A site prior to EF-G binding [17], and the mRNA is supposed to be in a “tensed” form since P codon AUG is fixed by tRNA^fMet^ with a minimum distance (7 nucleotides) between P codon (+1) and the core adenosine (−8) of SD sequence. Moreover, a non-cognate tRNA^fMet^, the unique tRNA used during complex preparation, is bound in the E site. The present structure of ribosome complex carries the E-codon AUG, which is originally translocated from the P site leading to 10 nucleotides, instead of 7 nucleotides, for the above distance, and thus results in a “relaxed” form of mRNA bound to the ribosome. Surprisingly, comparison of these two structures reveals no evident difference in the path of the mRNA in the ribosomal E site (Fig. 2). Even a non-cognate tRNA^fMet^ bound to the E site, the E-codon AAA of mRNA in “tensed” form (7 nucleotides for the distance aforementioned), still preserves the tendency to form interaction with anticodon of tRNA, which appears in contradiction with the early postulation that mRNA in “tensed” form might not be favorable for codon-anticodon interaction in ribosomal E site [15].

Interestingly, we found two ribosomal components in the present and previous structures [17], nucleotide G693 of 16S rRNA and Gly82 (at the tip of the β-hairpin loop 77–84) in ribosomal protein S7, are involved in a network of interaction between the first nucleotide of E-codon and the nucleotide immediately upstream of the E-codon, although the interaction is not identical (Fig. 1). In case of the present mRNA in a “relaxed” form, the interaction of G693 in 16S rRNA with the first nucleotide of E codon is consistent with the previous observation in the post-initiation complex [16]. The electron density map clearly shows that G693 still forms strong interactions with the first nucleotide of E-codon (Fig. S2), even under the mRNA in a “tensed” form as observed in the previous ribosome complex [17]. Particularly, the base stacking interaction between G693 and the first E-codon observed in the two structures prompts us to speculate that G693 is critical to facilitate E-codon positioning to form base pairs with anticodon of E-tRNA (Fig. 2). It is likely that the ribosome intrinsically preserves the potential to form codon-anticodon interaction in the E site, which could be evolutionally optimized.

### 3. An entire L1 Stalk Interacting with the Cognate E-tRNA

**Figure 3:**
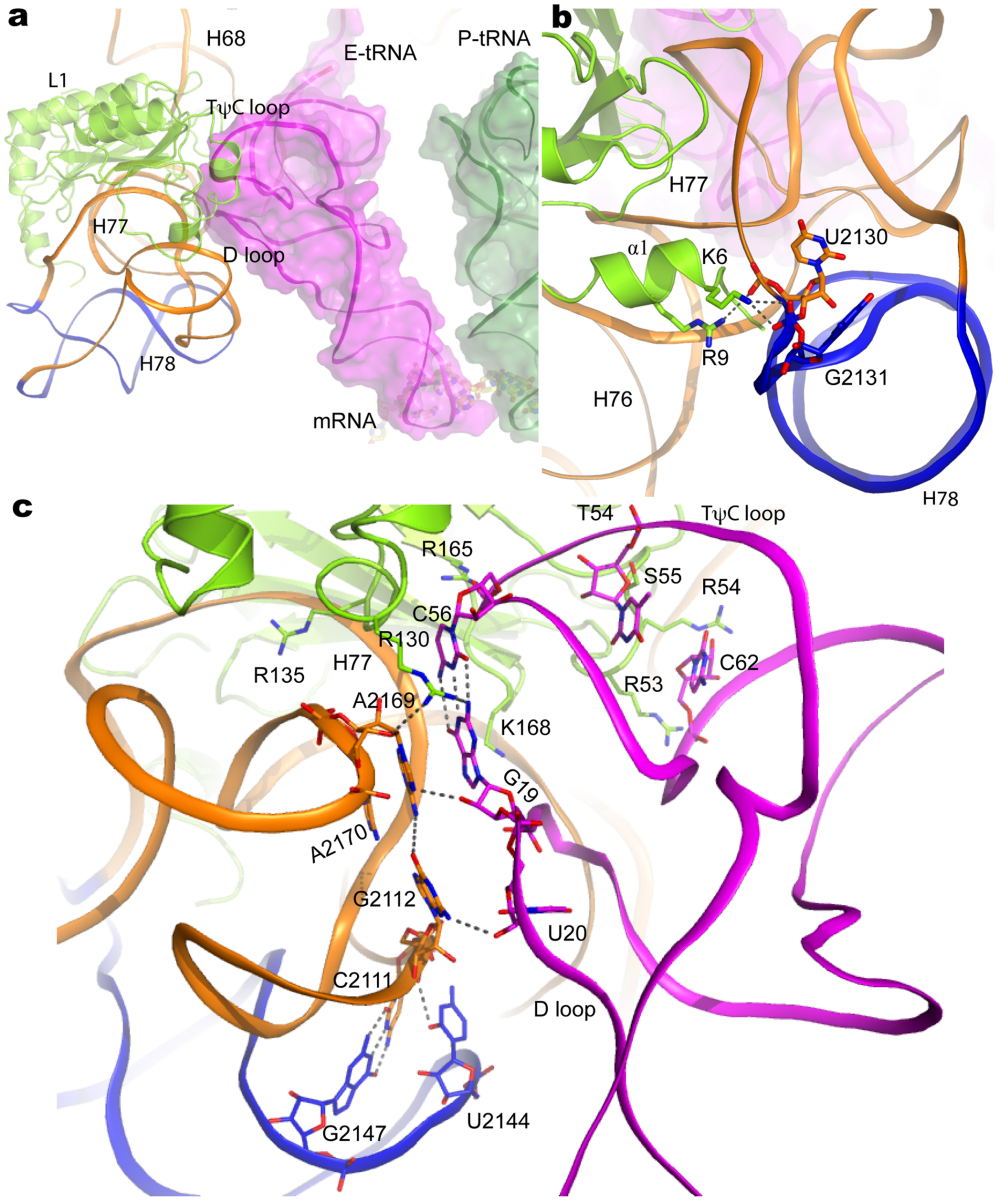
Interactions of L1 protein, L1 rRNA and the cognate E-tRNA. (a). A complete model of entire L1 stalk interacting with E-tRNA in 70S. The newly built H78 is colored blue. (b). Interactions of domain I of L1 with 23S rRNA. (c). The detailed interactions among L1 stalk (L1 protein and L1 rRNA) with E-tRNA.

The L1 stalk, consisting of ribosomal protein L1, helices 76–78 of 23S rRNA (L1 rRNA), is believed to be in contact with a cognate E-tRNA, which is associated with the function of the L1 stalk in hybrid state formation, tRNA movement and release [27]. Here we present the structure of the entire ribosomal L1 stalk (L1 protein and L1 rRNA) bound to a cognate E-tRNA (Fig. 3). Protein L1, comprising two extremely flexible domains I (N- and C-termini) and II (residues 70–160), resembles a clamp to hold L1 rRNA (region 2120–2127 in H77) (Fig. 3A). Domain I forms extensive contacts with H76 and H77 of 23S rRNA. The β-strands in domain I, face the groove of H77 to establish direct interaction, which is the most important contribution to the interaction of protein L1 with the ribosome. The N-terminal loop and helix α1 project deep into the pocket surrounded by H76–78 (Fig. 3B). Notably, the interactions of Lys6-G2131 and Arg8-U2130 facilitate the stabilization of the region 2131–2158 (colored blue in Fig. 3B) in H78 of the mobile L1 rRNA, which was found disordered in most 70S structures [19], [28].

Compared with domain I in L1 protein, domain II has fewer interactions with rRNA, suggesting more flexibility, which is supported by the simulation data that domain II can move independently of the rest of the L1 stalk [27]. Loop residue Arg135 projects into the groove of H77 to make direct contact with rRNA. In close proximity to Arg135, Arg130 makes a network of interactions involving L1 protein, L1 rRNA and E-tRNA, the three components relevant to the function of L1 stalk (Fig. 3C). The guanidinium group of Arg130 makes strong bidentate interactions with ribose O4 of A2169 and N2 of G19 in the D-loop of tRNA, and the aromatic bases of A2169 and G19 are stacking (π–π interaction). Additionally, the N7 in A2169 forms a hydrogen bond with ribose O2 in G19. Furthermore, the interactions of A2169 with G2112 (L1 RNA) contacting U20 in the D-loop of tRNA, and G19 with C56 which is within hydrogen-bonding distance to Arg165 in domain I of L1 protein, broaden the network of interactions. Next to A2169 in L1 RNA, A2170 is involved in the stacking interaction between A2169 and G19.

Three sequential residues Arg53, Arg54, and Ser55 in the loop of domain I of L1 protein contact both strands of TψC loop, and in the close proximity, loop residue Lys168 contacts D loop in the E-tRNA. Notably, G19 and C56, located in the tips of D- and TψC loops, form a canonical Watson-Crick base pair (Fig. 3C), which is remarkably crucial for tRNA acylation and editing, mutation to disrupt this base pairing interaction results in abolishing aminoacylation activity [29]. Interestingly, the G19C/C56G variant does not show any difference in aminoacylation activity [30]. In combination with other mutations in the D and TψC loops of tRNA, it is proposed that the G19C/C56G variant retains the tertiary loop-loop interaction in the elbow region, thereby mediating the communications between the two domains of the L-shaped tRNA to correctly recognize the cognate aminoacyl-tRNA synthetase in aminoacylation and editing reactions [29]. Appropriate aminoacylation and editing for amino acid with the tRNA serves as the first step responsible for translation fidelity. It is, therefore, unsurprising that the base pair of G19 and C56 retains the tertiary structure in the outer region of tRNA elbow for L-shape based specificity to ribosome, which is conducted by the interactions observed here among L1 protein, L1 rRNA and E-tRNA. The positioning of these three components could be essential for efficient ejecting a deacylated tRNA from the E site and directing tRNA movement. Indeed, distortion of the L shape accompanying tRNA movement across the ribosomal surface leads to an evident decrease in translocation activity [31].

C2111, next to G2112 participating in the formation of the aforementioned interaction network, forms a Watson-Crick base pair with G2147, and a hydrogen bond with C2144 (Fig. 3C). These two nucleotides locate at H78 in which U2130 and G2131 form contacts with L1 N-terminus. Taken together, these interactions stabilize the mobile H78 of 23S RNA resulting in the visualization of a complete model of an entire L1 rRNA in 70S structure (Figs. 1 and 3).

### 4. The Conformational Change of the C-terminal Tail of Protein S13 Relevant to Translocation Coordination

**Figure 4:**
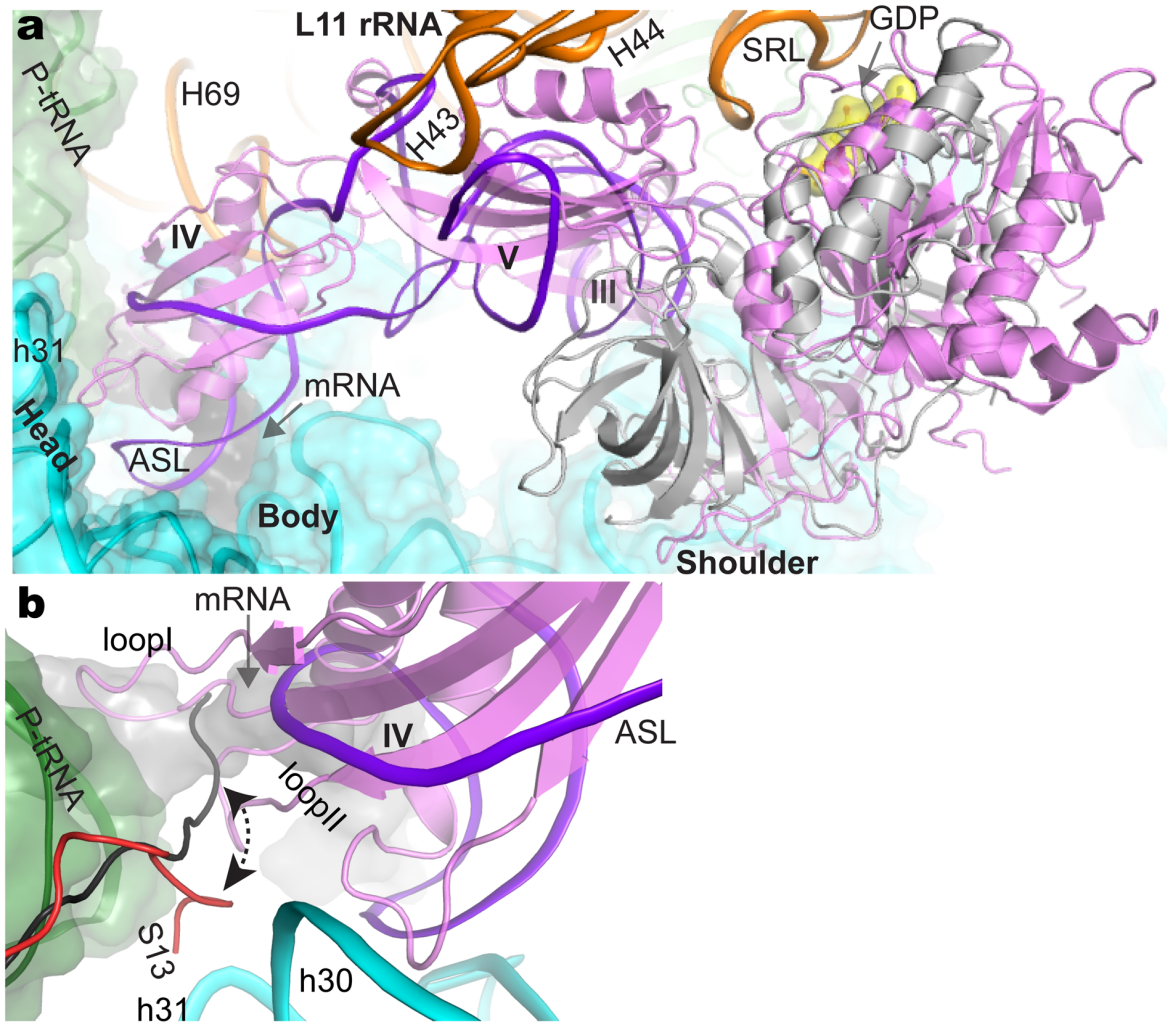
Structural comparison of EF-G with EF-Tu bound a distorted A-tRNA in the ribosome and conformational change of the C-terminal tail of ribosomal protein S13. The bound GDP in EF-G was shown as yellow surface model, indicated by arrow. The head, body, and shoulder domains of 16S rRNA (cyan surface model) are labeled. (a). Superposition of EF-Tu to EF-G in ribosome by fitting 23S rRNA. EF-G and EF-Tu complexed with tRNA are held in the same pocket, surrounded by 23S rRNA (sarcin-ricin loop SRL, L11 rRNA including H43 and H44, intersubunit bridge B2a H69), and 16S rRNA spanning both head (h31) and body (h18). (b). Conformational change of C-tail of protein S13. Protein S13 in the present structure and EF-Tu bound to ribosome are colored red and grey.

The structure of isolated EF-G is similar to the overall shape of the ternary complex of EF-Tu, tRNA and GTP analog (GDPNP) [32]. Interestingly, the comparison of EF-G and EF-Tu bound to tRNA in the ribosome shows some distinguishing differences. As expected, the two factors bind to the same pocket in the ribosome (Fig. 4A), the overall shape of the domains III-V of EF-G is a molecular mimicry of the distorted A-site tRNA which is bound to EF-Tu in the ribosome prior to accommodation, with domains III, IV, and V resembling the acceptor stem, the anticodon & D arm, and the T stem of tRNA, respectively. In contrast to the ASL of tRNA which forms base pairing with the A codon, the tip of domain IV (loops I and II) occupies a distinct position in close proximity to the P-tRNA, without direct interaction with the A-site codon (A-codon). The different positions of the tip of domain IV and the ASL in the decoding center result in a large conformational change of the C-terminal tail of S13 between the two structures (Fig. 4B).

The N terminus of S13 makes direct contact with the 50S subunit through two distinct bridges (bridge 1A between S13 and H38 of the 23S rRNA and 1B between S13 and L35), while the C terminus normally projects into the decoding center to interact with the P-tRNA [19]. It is noted that Arg125 in the C-terminal tail of S13 is within hydrogen-bonding distance to the A-codon in the structure of EF-Tu and tRNA bound to the ribosome [33]. In the present structure, the C-terminal tail of S13 swings towards h31 of 16S rRNA, and is located ∼18 Å apart from the A-codon (Fig. 4B). Previous results have shown that S13 has an important function in translocation, very likely by coordinating tRNA movement from one location (the interface of ribosome) to another (tRNA in the decoding) [34]. The location of the C-terminal tail of S13 in the present structure is distinct to the 70S structures with tRNA or other factors in the A site, providing a structural basis for the role of S13 in coordinating translocation which is probably associated with the flexibility of its tail in the decoding center. It is plausible that the positioning of S13 tail in different location may coordinate the movement of A/P tRNA to P/E tRNA in the process of translocation catalyzed by EF-G. Modification or truncation (C-terminal tail) of S13 may disrupt this communication network critical to translocation [34]. Indeed, *in vitro* assay of S13-deleted ribosome shows evident deficiency in translocation [35].

### 5. Ribosomal Protein L6: Interaction with EF-G Revealing its Particular Positioning in Ribosome

**Figure 5:**
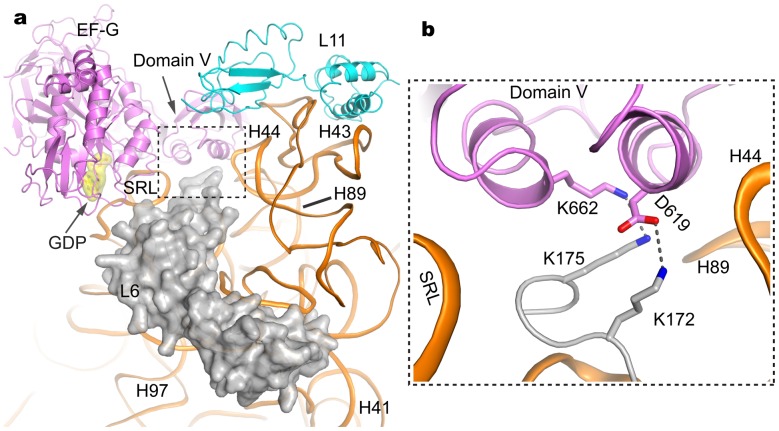
The positioning of protein L6 and its interaction with EF-G. (a). L6 and elements of EF-G in the vicinity of the L11 rRNA region (H43–44) and the SRL. L6 was represented as surface model in grey. (b). Overlarge view of detailed interactions of L6 and EF-G involving L11 rRNA and SRL.

Ribosomal protein L6 comprises N- and C-domains assembling to an elongated L-shaped structure that clamps the 23S rRNA (Fig. 5A). Upon EF-G binding, the C-terminal residues Arg170 −Gly177 of L6 are ordered, and deeply project into the pocket surrounded by domain V of EF-G, GTPase associated center (GAC) components (sarcin-ricin loop SRL and L11 RNA H44), and H89 of 23S RNA (Fig. 5A and 5B). It is noted that the C-terminal tail of L6 contains three conserved lysine residues, of which two residues (Lys172 and Lys175) are involved in contacting the domain V of EF-G. The side chain of lys172 in L6 forms a hydrogen bond with the side chain of Asp619 in EF-G, and both side chains of Lys175 in L6 and Lys662 in EF-G are within hydrogen-bonding distance (3.4 Å), which could rationalize the importance of the lysine motif in translation by enhancing EF-G binding [36]. Conformational change triggered by GTP hydrolysis would likely to be transmitted to domains IV and V via domain III leading to EF-G release [37]. Domain V of EF-G and L6 together make bilateral contacts with SRL and L11 RNA, bridging the two GAC components to form a network of interactions that may function as a sensor to probe and process signaling in the ribosome for translational factor binding and GTPase activation (Fig. 5B). Early study showed that position 637 in EF-G could be crosslinked to L6 with higher efficiency in the pre than in the posttranslocational state [38], suggesting that the interaction between EF-G and L6 could change during translocation.

It was reported that L6 mutations, supposed to be a reading frame shift and premature termination in the C-terminal half, would result in fusidic acid resistance [39]. In addition to the direct interaction of the C-terminus of L6 with EF-G (Fig. 5B), a role of L6 in stabilizing the positioning of the SRL and L11 RNA, which in turn interacts with EF-G and “locks” it in the presence of fusidic acid, appears to be the reason for fusidic acid resistance in L6 mutation. Interestingly, these L6 mutations are partly resistant to gentamycin, kanamycin, and streptomycin [36]. Moreover, L6 mutations have effects on the accuracy of translation [40]. To rationalize these data, a plausible explanation involves the positioning of L6 in the ribosome, in particular the positioning of its C-terminal tail which bridges the SRL and L11 RNA region to form a compact ribosome GAC essential for all phases of protein translation.

## Discussion

Using ribosome lacking L9, we obtained a crystal form belonging to space group P2_1_, which enable us to determine the structure of EF-G bound to ribosome, in which a cognate P-tRNA and a non-cognate E-tRNA were presented [17]. To address the question of base pairing between codon and anticodon in the E site where a non-cognate tRNA is often observed, we prepared new ribosome complex by EF-G catalyzing the translocation of cognate tRNAs (together with mRNA codon) from A and P to P and E sites. Here, we report the structure of this ribosome complex with two cognate P- and E-tRNAs.

### 1. The Cognate tRNA in Both E and A Sites: Implications for Structural Collaboration

Since ribosomal E site was proposed three decades ago [6], its function is still a matter of controversy. Nevertheless, it is generally agreed that it has a role in translational frame maintenance by a number of groups using both *in vitro* and *in vivo* analysis [12], [41], [42]. To maintain translational frame, stable binding between ribosome and mRNA and tRNA substrates has to take place. Therefore, a cognate E-tRNA in the elongation complex is likely to have a role in frame maintenance, and a plausible explanation is that the anticodon of deacylated tRNA translated into E site is involved in base pairing with mRNA [43]. However, the evidence available is limited. Here we report for the first time the structure of a cognate E-tRNA stacking with an entire L1 stalk in the 70S and observe codon-anticodon paring for the first two nucleotides of E-codon (Fig. 1). Surprisingly, the third position codon (G) does not make a direct contact with the anticodon (C), but does so through one water molecule via bidentate hydrogen bonds. In the A site, codon-anticodon paring at the third position (“wobble”) is less strict and near cognate is acceptable, rather than the first two positions where exact base pairs are indispensible. Such interactions provide a delicate balance between the energy derived from binding of a cognate tRNA and the combined energy required for distortions in the tRNA, EF-Tu and the 30S subunit that enable GTP hydrolysis, so that proper decoding could be achieved [24], [33]. Similarly, the interactions of codon-anticodon observed here, likely depict the nature of the ribosome with a well-balanced interaction of a cognate E-tRNA for mRNA codon during the elongation cycle. It is well known that three nucleotides of P codon are involved in codon-anticodon pairing. The mechanism of evolution with regards to the codon-anticodon pairing for the three ribosomal sites, and whether the similarity of A- and E-tRNAs interacting with their codons, base pair for the first two positions and less strict interaction for the third, is relevant to tRNA incorporation and rejection, both remain elusive.

### 2. The Dynamics of Ribosomal L1 Stalk Stabilized by EF-G Binding

The L1 stalk, one of the most dynamic components of the ribosome, is found in three states, so-called “open” with vacant E site, “half-closed” with a non-anticodon E-tRNA, and “closed” with a hybrid P/E-tRNA [44]. This dynamic feature is believed to be a prerequisite in assisting tRNA/mRNA movement [27]. However, the extreme flexibility is a major obstacle for obtaining the entire structure of the L1 stalk. Unexpectedly, the L1 stalk was stabilized upon EF-G occupying the A site, although there appears to be no direct interactions, thus has enabled us to obtain a complete model for the entire L1 stalk and the interacting cognate E-tRNA, which represents an authentic ribosome elongation complex (Figs. 1 and 3). The L1 stalk and its interacting cognate E-tRNA in the present structure shifts towards the 50S body compared with that of 70S structure at 2.8 Å [19] (Fig. S3A). The main chain of Lys60 in protein L1 and A2169 in 23S rRNA move inwards by 16.6 and 18.1 Å, and the tip of the elbow of E-tRNA by ∼7 Å, respectively. In the case of the elongation factor P (EF-P) bound to ribosome [45], the L1 stalk moves much further to occupy the position of the cognate E-tRNA (Fig. S3B). The distance of Lys80 in the domain II of L1 to Ala50 in L5 appears to be ∼40 Å in the present structure, whereas these two residues are within interaction distance in the structure of ribosome with EF-P. Given that the conformation and location of L5 in ribosome is almost identical in both structures, the large conformational change observed can be completely ascribed to the movement of L1 stalk.

Very recently, the structures of ratcheting ribosome have become available: the structure of rotated *E. coli* ribosome either stabilized by ribosome recycling factor (despite lacking of protein L1) [46] or presented with RF3 [47]; and the structure of rotated *T. thermophilus* ribosome in the presence of RF3 [48]. Notably, the L1 stalks in these structures are in slightly more “closed” states compared with that in the present structure. Indeed, the L1 stalk observed in our structure with an authentically translocated tRNA in the E site is in a state between “half closed” and “closed” states (Fig. S3), in line with the previous report that the presence of translocated E-tRNA could trigger the fluctuations of L1 stalk between “open” and “closed” states [44]. We analyzed the crystal contact in two forms P2_1_ and P2_1_2_1_2_1_ obtained from L9 mutant and wild type 70S ribosomes, respectively, it seems that the L1 stalk is involved in the crystal contact in both forms, but the contacts are not identical. We can not completely rule out crystal contact that may contribute to the different conformation of L1 stalk and the stabilization. However, the difference of crystal contact in L1 stalk in the two forms and the impact resulting from this crystal contact appears to be quite limited. Taken together, our findings suggest a structural link between L1 stalk and EF-G binding, which could provide a molecular communication for their allosteric collaboration in directing tRNA movements proposed by real time smFRET [49].

## Supporting Information

Figure S1 **Structural comparison of three tRNAs in the E site.** The tRNAs from our previous complex (PDB: 2WRI, no codon-anticodon interaction), post-initiation complex (PDB: 2HGP, codon-anticodon pairing for the first nucleotide), and the present complex, are colored grey, marine, and magenta, respectively. Structure was fitting to the present complex by 16S rRNA. Two major conformational changes were observed at the ASL and D loop where interactions of ASL with E codon, as well D loop with L1 stalk are made in the present structure.
(TIF)

Figure S2 **Representative electron density from a 3 mF_O_–2 dF_C_ map contoured at 2.0 σ.** The refined models of G693 of 16S RNA, A-3 and A-2 of E codon are labeled. The interactions between G693 and A_-3_, are depicted as: dashed line is within hydrogen-bonding distance, solid line is within stacking distance.
(TIF)

Figure S3 **Conformational change of L1 stalk and E-tRNA.** Ribosomal protein L5 is colored prupleblue, with A50 shown in stick which makes interaction with K80 of L1 in the structure of EF-P bound to ribosome. The newly built 23S rRNA in L1 stalk (H78) is colored blue. (a), (b). Comparison of L1 stalk and E-site tRNA in the present structure with that of 2.8 Å structure (colored grey), and with that of EF-P bound structure (colored grey, but L1 colored yellow for obvious contrast).
(TIF)
