# Supplementary figures and images for: Crystal Structure of 70S Ribosome with Both Cognate tRNAs in the E and P Sites Representing an Authentic Elongation Complex
Source: PLoS One. 2013 Mar 19;8(3):e58829. doi: 10.1371/journal.pone.0058829 (PMC3602588; doi:10.1371/journal.pone.0058829)

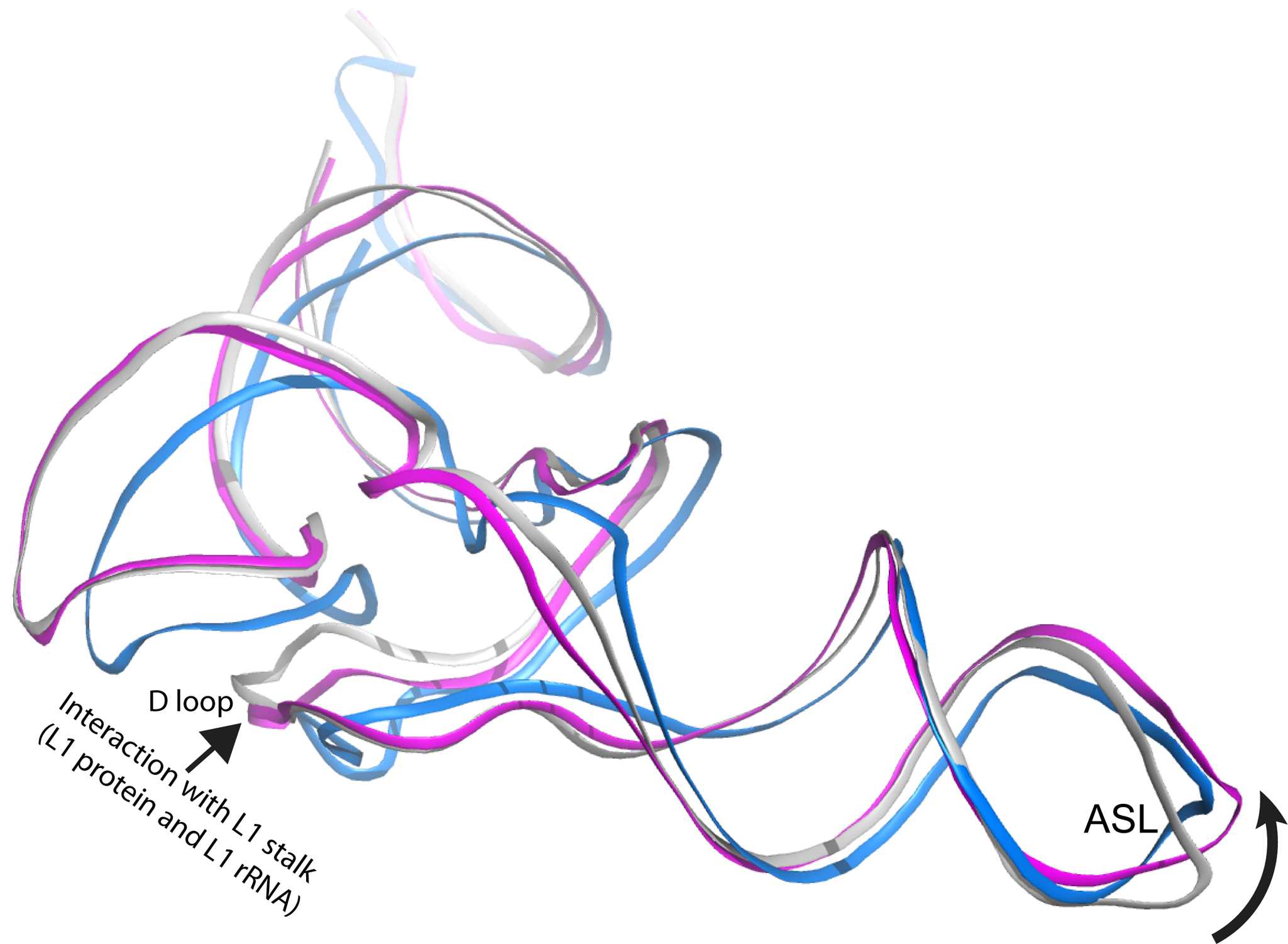

Supplement: Figure S1 — Structural comparison of three tRNAs in the E site. The tRNAs from our previous complex (PDB: 2WRI, no codon-anticodon interaction), post-initiation complex (PDB: 2HGP, codon-anticodon pairing for the first nucleotide), and the present complex, are colored grey, marine, and magenta, respectively. Structure was fitting to the present complex by 16S rRNA. Two major conformational changes were observed at the ASL and D loop where interactions of ASL with E codon, as well D loop with L1 stalk are made in the present structure. (TIF) [file pone.0058829.s001.tif]

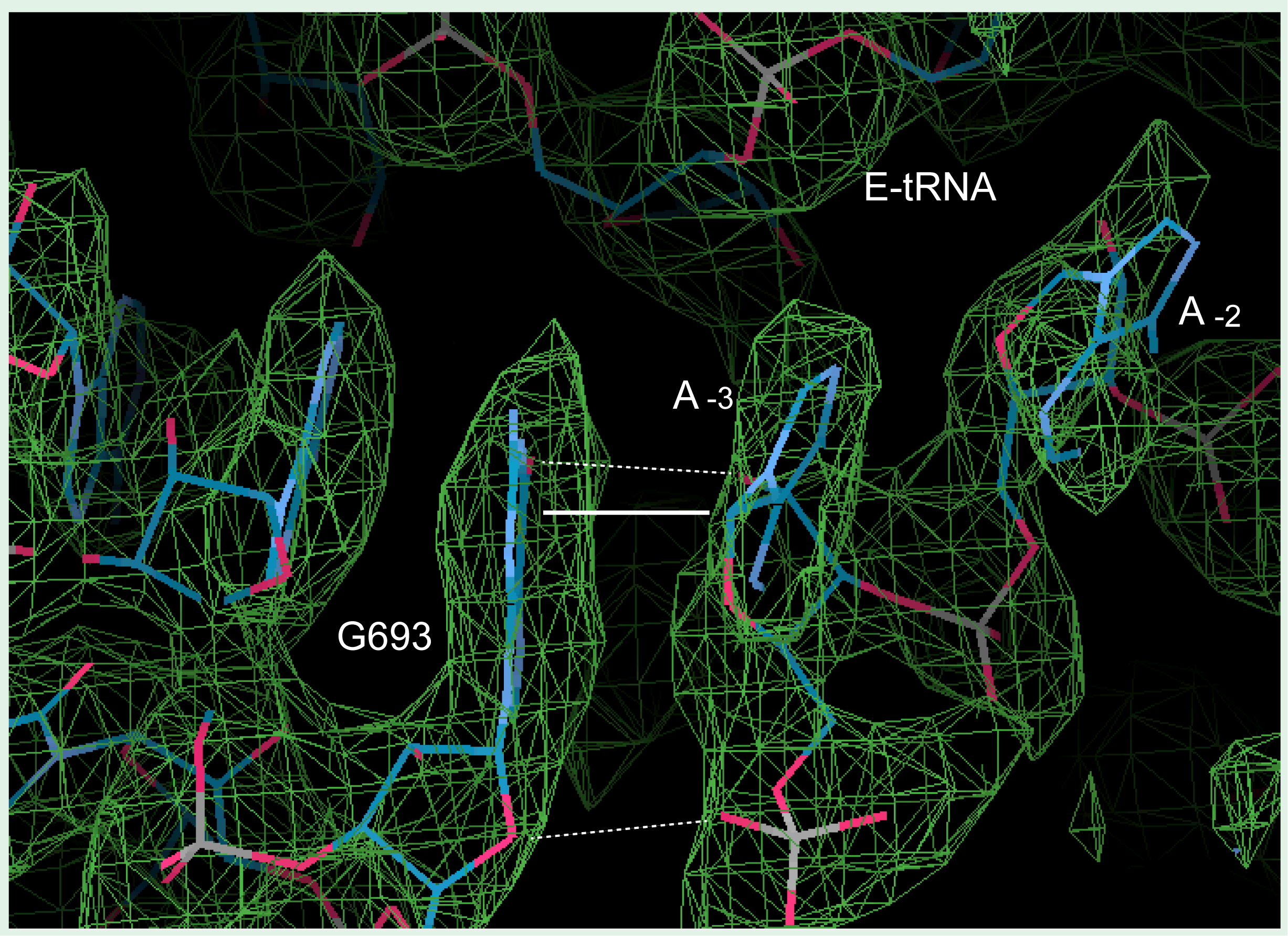

Supplement: Figure S2 — Representative electron density from a 3 mFO–2 dFC map contoured at 2.0 σ. The refined models of G693 of 16S RNA, A-3 and A-2 of E codon are labeled. The interactions between G693 and A-3, are depicted as: dashed line is within hydrogen-bonding distance, solid line is within stacking distance. (TIF) [file pone.0058829.s002.tif]

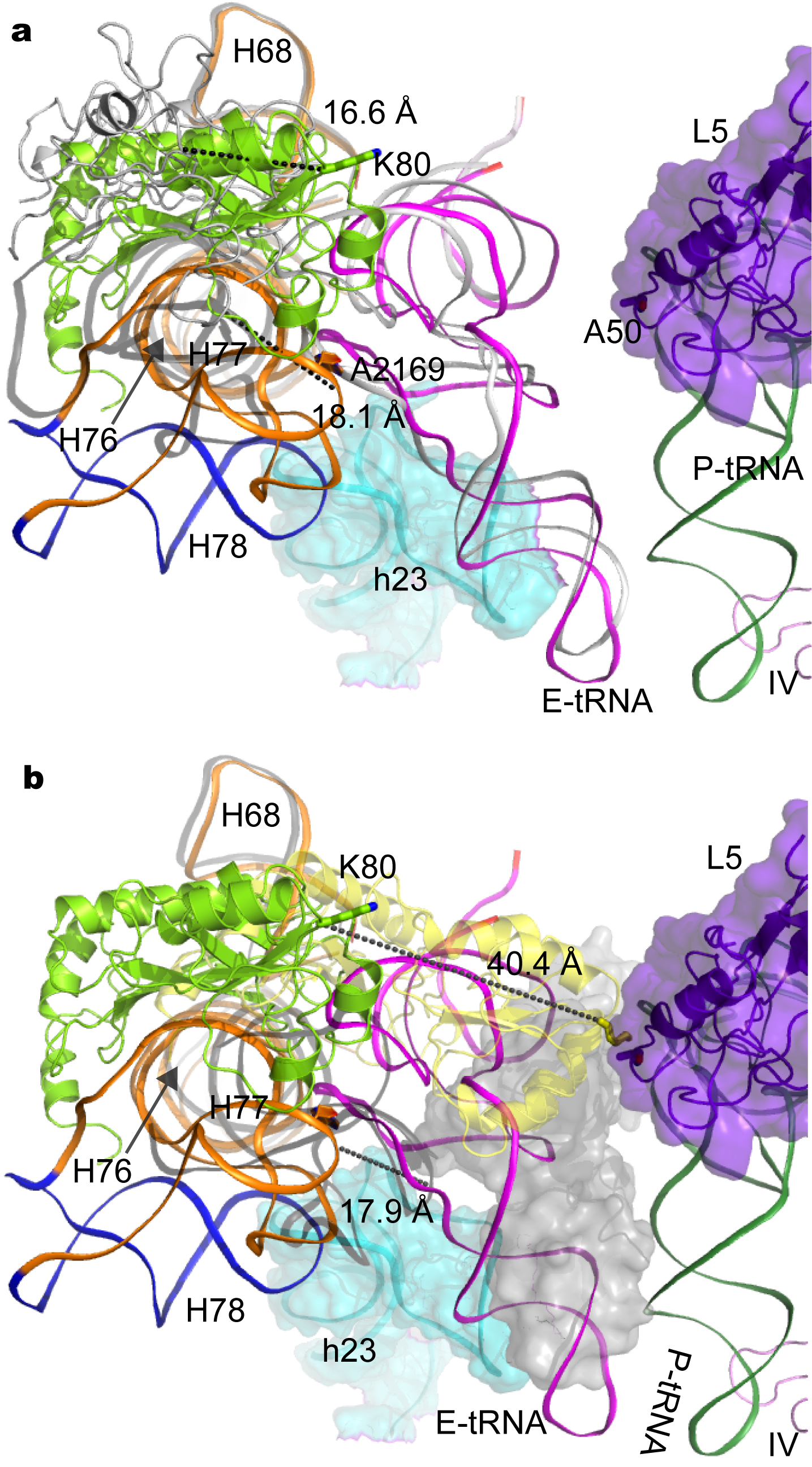

Supplement: Figure S3 — Conformational change of L1 stalk and E-tRNA. Ribosomal protein L5 is colored prupleblue, with A50 shown in stick which makes interaction with K80 of L1 in the structure of EF-P bound to ribosome. The newly built 23S rRNA in L1 stalk (H78) is colored blue. (a), (b). Comparison of L1 stalk and E-site tRNA in the present structure with that of 2.8 Å structure (colored grey), and with that of EF-P bound structure (colored grey, but L1 colored yellow for obvious contrast). (TIF) [file pone.0058829.s003.tif]
